# Prevalence of diarrhoea and risk factors among children under five years old in Mbour, Senegal: a cross-sectional study

**DOI:** 10.1186/s40249-017-0323-1

**Published:** 2017-07-06

**Authors:** Sokhna Thiam, Aminata N. Diène, Samuel Fuhrimann, Mirko S. Winkler, Ibrahima Sy, Jacques A. Ndione, Christian Schindler, Penelope Vounatsou, Jürg Utzinger, Ousmane Faye, Guéladio Cissé

**Affiliations:** 1 Swiss Tropical and Public Health Institute, P.O. Box, CH-4002 Basel, Switzerland; 2 University of Basel, P.O. Box, CH-4003 Basel, Switzerland; 3 Département de Géographie, Université Cheikh Anta Diop de Dakar, BP 5005 Dakar, Senegal; 4 Centre de Suivi Ecologique, BP 15 532 Dakar-Fann, Senegal; 5 Département de Biologie Animale, Université Cheikh Anta Diop de Dakar, BP 5005 Dakar, Senegal

**Keywords:** Children under five year-old, Cross-sectional survey, Diarrhoea, Multivariable logistic regression, Risk factor, Senegal

## Abstract

**Background:**

Diarrhoeal diseases remain an important cause of mortality and morbidity among children, particularly in low- and middle-income countries. In Senegal, diarrhoea is responsible for 15% of all deaths in children under the age of five and is the third leading cause of childhood deaths. For targeted planning and implementation of prevention strategies, a context-specific understanding of the determinants of diarrhoeal diseases is needed. The aim of this study was to identify risk factors of diarrhoeal diseases in children under the age of five in Mbour, Senegal.

**Methods:**

Between February and March 2014, a cross-sectional survey was conducted in four zones of Mbour to estimate the burden of diarrhoeal diseases (i.e. diarrhoea episodes in the 2 weeks preceding the survey) and associated risk factors. The zones covered urban central, peri-central, north peripheral and south peripheral areas. Overall, 596 households were surveyed by a questionnaire, yielding information on sociodemographic, environmental and hygiene behavioural factors. Univariable and multivariable logistic regression analyses were used to identify risk factors associated with the occurrence of diarrhoea.

**Results:**

The reported prevalence of diarrhoea among children under the age of five during the 2 weeks preceding the survey was 26%. Without adjustment, the highest diarrhoea prevalence rates were observed in the peri-central (44.8%) and urban central zones (36.3%). Multivariable regression revealed significant associations between diarrhoeal diseases and unemployment of mothers (adjusted odds ratio [a*OR*] = 1.62, 95% confidence interval [*CI*]: 1.18–2.23), use of open bags for storing household waste (a*OR* = 1.75, 95% *CI*: 1.00–3.02), evacuation of household waste in public streets (a*OR* = 2.07, 95% *CI*: 1.20–3.55), no treatment of stored drinking water (a*OR* = 1.69, 95% *CI*: 1.11–2.56) and use of shared toilets (a*OR* = 1.69, 95% *CI*: 1.11–2.56).

**Conclusion:**

We found a high prevalence of diarrhoea in children under the age of five in Mbour, with the highest prevalence occurring in the central and peri-central areas. These findings underscore the need for public health interventions to alleviate the burden of diarrhoea among vulnerable groups. Promotion of solid waste disposal and reduction of wastewater exposure should be implemented without delay.

**Electronic supplementary material:**

The online version of this article (doi:10.1186/s40249-017-0323-1) contains supplementary material, which is available to authorized users.

## Multilingual abstracts

Please see Additional file 1 for translations of the abstract into the five official working languages of the United Nations.

## Background

Diarrhoeal diseases remain among the most common causes of mortality and morbidity in children, particularly in low- and middle-income countries (LMICs). In 2013, of the 6.3 million children worldwide who died before they reached their fifth birthday, about half (3.2 million) died from infectious diseases, with diarrhoea killing more than 500,000 children [1]. By 2030, it is estimated that 4.4 million children under the age of five will die from infectious diseases annually and that 60% of those deaths will occur in sub-Saharan Africa [1]. Diarrhoea accounts for an estimated 3.6% of the global burden of disease, as expressed in disability-adjusted life years (DALYs) [2]. Although mortality from diarrhoea has declined considerably over the past 25 years globally, morbidity from diarrhoea in sub-Saharan Africa has not, as risk factors related to inadequate water, sanitation and hygiene (WASH), insufficient promotion of breastfeeding and malnutrition remain unacceptably high [3]. The rapid growth of African cities and associated overcrowding has been linked to outbreaks of diarrhoea, with children under the age of five among the most affected [4].

In Senegal, the Ministry of Health (MoH) lists diarrhoea as the third leading cause of mortality and the second leading cause driving caregivers of children under 5 years old to seek medical consultation [5]. In 2013, according to Liu and colleagues, the total number of deaths among children under the age of five in the country was 28,648, with 1866 deaths (15%) due to diarrhoeal diseases [1, 6]. In 2011, the Senegalese Demographic and Health Survey (DHS) reported that one in five children under the age of five suffered from diarrhoea during the 2 weeks preceding the survey (21%) [7]. The 2014 DHS showed that prevalence of diarrhoea in this age group remained at the same level (19%) [8].

Lack of access to clean water and improved sanitation is a major issue in the urban coastal area of Senegal, where 13.5 million people reside, accounting for 45% of the national population [9]. Rapid urbanisation in sub-Saharan Africa (including Senegal) over the past few decades has resulted in disorganised urban landscapes, where populations live in crowded conditions [10, 11]. Urbanisation not only occurs in capital cities of LMICs, but also in secondary cities [12]. Indeed, half of the anticipated urban population increase in the coming years is expected to occur in secondary African cities and in smaller cities that connect the rural hinterlands of the sub-region [13–15]. The urbanisation trends in secondary cities merit focused attention due to the particular weaknesses and vulnerabilities of those contexts.

In Mbour, a secondary coastal city in Senegal, the population has grown from approximately 100,000 in 1988 to more than 220,000 in 2014 [16]. However, unofficial estimations by Mbour municipality leaders suggest that the current population might be as high as 700,000, which is far above the projection for 2014 made by the National Agency of Statistics and Demography (ANSD) on the basis of 2002 census data. This massive increase in Mbour’s population has also resulted in a spatial extension of 56% [17], the spread of urban slums and a lack of basic services pertaining to WASH and solid waste removal [18, 19]. These conditions create a high risk of water-borne and gastrointestinal diseases, including diarrhoea [20]. Not surprisingly, Mbour was among the ten health districts in Senegal characterised by high numbers of diarrhoeal cases. To better understand the determinants of diarrhoea in this urban setting, data are needed to better understand the local epidemiology. Such data will be valuable for designing and implementing intervention and prevention strategies to reduce morbidity due to diarrhoea at the community level.

Here, we provide an overview of diarrhoea prevalence and risk factors among children under 5 years old in Mbour. Specifically, we determined the prevalence of self-reported diarrhoea and identified key risk factors among young children in this secondary city of Senegal.

## Methods

### Study area

**Fig. 1:**
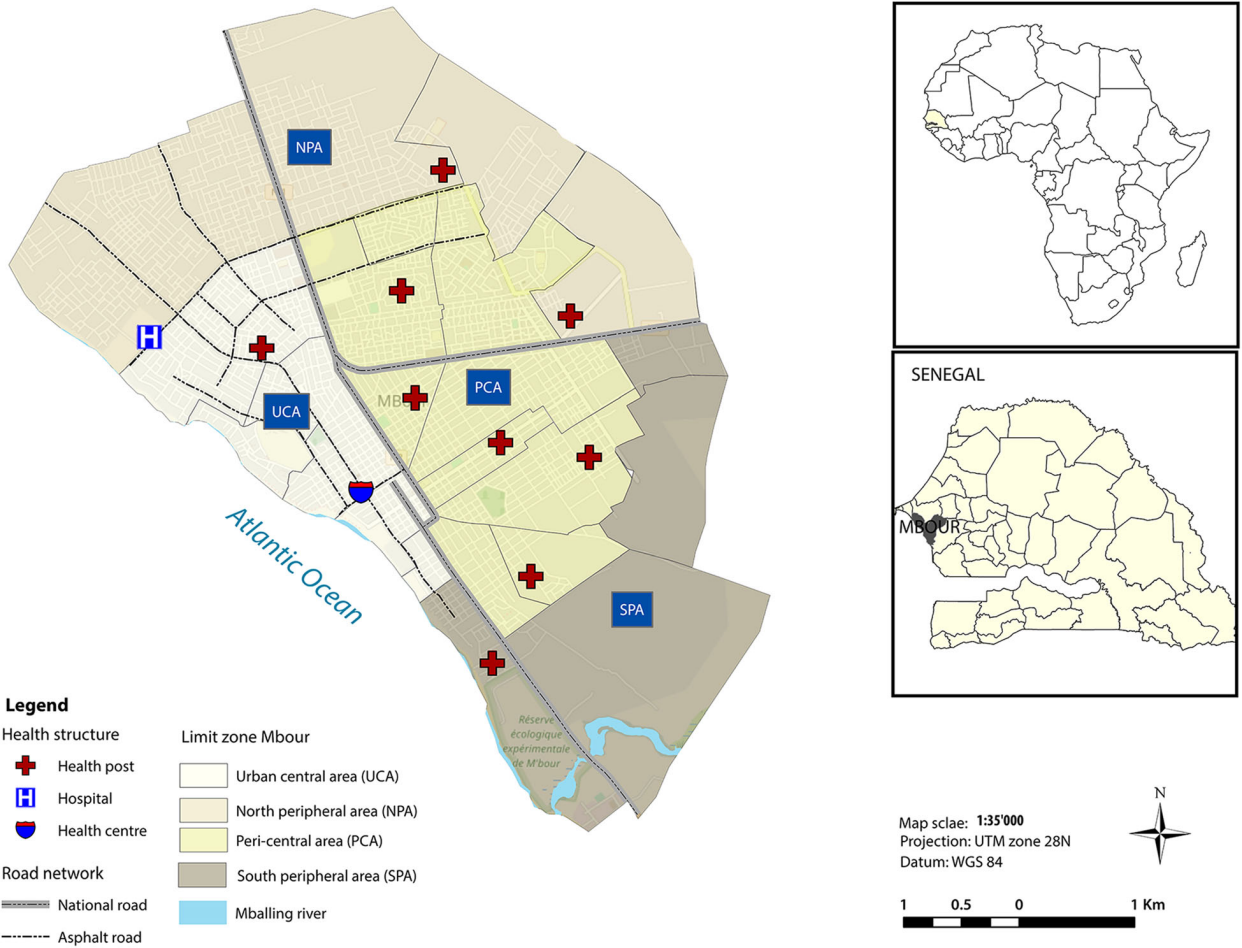
Map showing Mbour in Senegal and the location of the four different zones

The study was conducted in Mbour, a secondary coastal city in Senegal on the edge of the Atlantic Ocean (Fig. 1). The city lies approximately 19 m above sea level in the region of Thiès in western Senegal (latitude 14°41′6″N and longitude 16°96′9″W), about 80 km south of the capital, Dakar. Administratively, the city is divided into 25 neighbourhoods. Mbour’s health system consists of a hospital, a health centre and 10 health posts. The study area was stratified into four zones according to specific characteristics, namely: (i) urban central area (UCA); (ii) peri-central area (PCA); (iii) north peripheral area (NPA); and (iv) south peripheral area (SPA). The PCA, UCA, NPA and SPA cover areas of 7, 4, 8 and 6 km^2^, respectively. The respective population sizes are 98,126, 48,011, 53,894 and 28,259.

The UCA is the original core of the city and includes the central neighbourhoods, the historic district and commercial centre, all with a type of traditional habitat. It is characterised by poor sanitation, high population density and overcrowding and is inhabited mostly by fishermen. The PCA is the first peri-urban area of the city, composed of regular and irregular neighbourhoods like *Baye Deuk,* an informal neighbourhood surrounding the core. The NPA and SPA comprise new neighbourhoods or peripheral neighbourhoods. The NPA is marked by a modern habitat and better access to basic social services in some neighbourhoods, while the SPA is characterised by straw houses, a precarious socioeconomic status and a lack of access to safe drinking water. Most of the population in the SPA and NPA use water from traditional wells. These four zones were chosen to compare the prevalence and risk factors of diarrhoea related to living conditions, socioeconomic status, drinking water sources, sanitation facilities, hygiene and education levels.

### Outcome definition

The primary outcome variable was the occurrence of diarrhoea during the 2-week period preceding the survey interview. For this purpose, the DHS definition of diarrhoea was applied, i.e. having three or more loose or liquid bowel movements over a 24-h period, as reported by the mother or caregiver of the child, at any point during the 2 weeks preceding the interview [8]. This definition is in line with that of the World Health Organization (WHO) [21].

### Household survey and study population

A cross-sectional study was carried out between 2 February and 8 March 2014. As the prevalence of diarrhoea in Mbour had not been investigated in previous studies, we assumed an average prevalence rate of 26%, based on data obtained from the DHS and National Survey on Food Security and Nutrition (ENSAN), representing the Thiès region where Mbour is located [7, 22]. The sample size (*n*) was calculated to achieve sufficient precision in estimating the prevalence of diarrhoea among children under 5 years, using the following formula: *n* = *Z* ^*2*^ × p *× (1 -* p*)* × *c/r* ^*2*^ where: *Z* equals 1.96, p is the estimated prevalence of diarrhoea among children under 5 years (assumed to be 26%), *r* is the accepted margin of error (assumed to be 5%) and *c* is the design effect accounting for clustering of the data within households. Assuming *c* = 2, a sample size of 600 households was calculated. Having divided the city into four zones (i.e. PCA, UCA, NPA and SPA), we determined the sample size of households proportionally to the population for each zone. We randomly selected clusters of equal size (25 households) within each zone. Overall, we surveyed 24 clusters. These clusters were defined by the smallest administrative units, known as “district census”. The final sample size was 596 households.

Households were eligible for inclusion in the survey if the following criteria were met: (i) presence of mother or caregiver; and (ii) presence of at least one child under the age of five. A structured questionnaire was administered for data collection. The questionnaire was inspired by the Multiple Indicators Cluster Survey (MICS) and by DHS questionnaires related to diarrhoea. The French language version of the questionnaire was translated into local language and then translated back into French to ensure accuracy. Four experienced investigators conducted interviews in the local language or in French, using a paper-based questionnaire. Investigators were trained to administer the interview, to follow data quality assurance procedures and to adhere to principles of ethical conduct in human research. The survey questionnaire was pre-tested in a neighbourhood of Mbour that was not otherwise considered, to ensure that questions were properly understood by the local communities. In the pilot study, ten households were interviewed and any observed shortcomings in the instruments were corrected before the start of data collection. The pre-test also provided crucial information on the validity and usefulness of the data collected.

Independent variables included the following: (i) socio-demographic information (zone, mother’s age, family size, number of children under the age of five in the household, mother’s or caregiver’s educational level); (ii) socioeconomic status (occupation); (iii) environmental and behavioural indicators (availability of a toilet, type of toilet, drinking water source, solid waste and wastewater disposal, personal hygiene); and (iv) occurrence of diarrhoea. Socioeconomic status of the households was classified as either “richest”, “middle” or “poorest”, based on a cumulative standardised assets score, which was calculated using principal component analysis. All asset variables considered were dichotomous (e.g. presence or absence of radio).

### Statistical analysis

Statistical analysis was performed in Stata version 13.0 (Stata Corporation; College Station, United States of America). Descriptive statistics were used to summarise the study variables. Associations between outcome and independent variables were expressed by their odds ratios (*OR*s) and 95% confidence intervals (*CI*s). Mixed univariable and multivariable logistic regression models with random intercepts for households were used to quantify the effects of the risk factors on the diarrhoea outcome and to compare differences in diarrhoea prevalence between the zones. Models were compared using likelihood ratio tests (LRTs). To reduce the number of parameters and to improve precision of the estimates of the final models, we only considered variables that had a *P*-value below 0.2 in the univariable analysis (using LRT) for the multivariable analysis. Statistical significance was defined at the level of 5%.

## Results

### Sociodemographic characteristics of the surveyed households

**Fig. 2:**
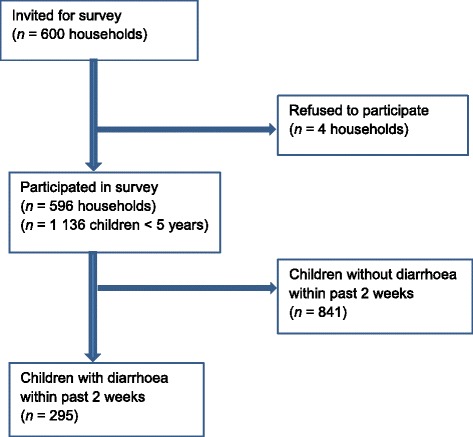
Summary of surveyed household and results of reported diarrhoea cases by age categories in Mbour, Senegal, 2014

A total of 1136 children under the age of five (50.8% males) from 596 households participated in the survey conducted in four zones of Mbour, in early 2014 (Fig. 2). Demographic and socioeconomic characteristics of the surveyed households are summarised in Table 1. The median age of the respondents was 30 years. Parental ages presented in Table 1 are for those parents with complete questionnaire results. Most of the mothers were married (93.5%; *n* = 275). The primary occupation was housewife (56.6%; *n* = 167). Almost half of the mothers had no formal education (45.6%; *n* = 272). The mean household size was 8.7 (standard deviation (*SD*): 4.8) individuals.

**Table 1:** Characteristics of the households surveyed (*n* = 596) in four zones of Mbour, Senegal, in early 2014

Variables	UCA (*N* = 175) *n* (%)	PCA (*N* = 324) *n* (%)	NPA (*N* = 47) *n* (%)	SPA (*N* = 50) *n* (%)	Overall (*N* = 596) *n* (%)	*P*-value
Caretaker characteristics						
Age in years						0.065
< 25	24 (15.9)	47 (18.9)	20 (22.5)	8 (19.5)	99 (18.7)	
25–29	39 (25.8)	52 (20.9)	13 (14.6)	2 (4.9)	106 (20.0)	
30–39	55 (36.4)	100 (40.1)	34 (38.2)	15 (36.6)	204 (38.5)	
≥ 40	33 (21.8)	50 (20.1)	22 (24.7)	16 (39.0)	121 (22.8)	
Educational level						**<0.001**
Never went to school	67 (38.3)	133 (48.5)	43 (44.3)	29 (58.0)	272 (45.6)	
Primary school	81 (46.3)	70 (25.6)	32 (33.0)	9 (18.0)	192 (32.2)	
Secondary education or higher^a^	27 (15.4)	71 (25.9)	22 (22.7)	12 (24.0)	132 (22.2)	
Household characteristics						
Number of household members						**<0.001**
< 5 members	25 (14.3)	43 (15.7)	20 (20.6)	6 (12.0)	94 (15.7)	
5–7 members	31 (17.7)	103 (37.6)	48 (49.5)	14 (28.0)	196 (32.9)	
8–10 members	40 (22.9)	70 (25.5)	19 (19.6)	15 (30.0)	144 (24.2)	
≥ 11 members	79 (45.1)	58 (21.2)	10 (10.3)	15 (30.0)	162 (27.2)	
Number of children <5 years in the house						**<0.001**
< 2	64 (36.6)	129 (47.1)	52 (53.6)	24 (48.0)	269 (45.1)	
2 to 3	77 (44.0)	126 (46.0)	42 (43.3)	25 (50.0)	270 (45.3)	
4+	34 (19.4)	19 (6.9)	3 (3.1)	1 (2.0)	57 (9.6)	
Socio-economic status						**<0.001**
Poorest	9 (5.2)	89 (32.5)	24 (24.7)	26 (52.0)	148 (24.9)	
Middle	55 (31.6)	54 (19.7)	24 (24.7)	16 (32.0)	149 (25.0)	
Richest	110 (63.2)	131 (47.8)	49 (50.5)	8 (16.0)	298 (50.1)	
Drinking water sources						**<0.001**
Tap water in the house	146 (83.4)	153 (55.8)	63 (64.9)	6 (12.0)	368 (61.7)	
Public tap	24 (13.7)	95 (34.7)	20 (20.6)	13 (26.0)	152 (25.5)	
Well water	2 (1.1)	9 (3.2)	13 (13.4)	27 (54.0)	51 (8.6)	
Others^b^	3 (1.7)	17 (6.2)	1 (1.0)	4 (8.0)	25 (4.2)	
Water storage						**<0.001**
No	67 (38.3)	58 (21.2)	38 (39.2)	18 (36.0)	181 (30.4)	
Yes	108 (61.7)	216 (78.8)	59 (60.8)	32 (64.0)	415 (69.6)	
Toilet availability						<0.001
No	2 (1.1)	6 (2.2)	1 (1.0)	7 (14.0)	16 (2.7)	
Yes	173 (98.9)	268 (97.8)	96 (99.0)	43 (86.0)	580 (97.3)	
Type of toilet facilities						<0.001
Sewer	4 (2.3)	2 (0.7)	1 (1.0)	0	7 (1.2)	
Toilet with pit	166 (94.9)	227 (84.7)	91 (94.8)	38 (88.4)	522 (90.0)	
Traditional latrine	3 (1.7)	39 (14.6)	4 (4.2)	5 (11.6)	51 (8.8)	
Toilet shared with others households						**<0.001**
No	112 (64.7)	145 (54.1)	66 (68.8)	35 (81.4)	358 (61.7)	
Yes	61 (35.3)	123 (45.9)	30 (31.2)	8 (18.6)	222 (38.3)	
No kitchen available in the house	52 (29.7)	102 (37.2)	21 (21.6)	26 (52.0)	201 (33.7)	**<0.001**
Behavioural characteristics						
Duration of storage						0.233
One day	54 (57.5)	108 (54.5)	32 (56.1)	14 (43.7)	208 (54.6)	
Two days	22 (23.4)	45 (22.7)	12 (21.1)	15 (46.9)	94 (24.7)	
Three days	10 (10.6)	32 (16.2)	8 (14.0)	1 (3.1)	51 (13.4)	
More than four three days	8 (8.5)	13 (6.6)	5 8.8)	2 (6.2)	28 (7.3)	
Treatment water stored						**<0.001**
Yes	21 (12.0)	36 (13.1)	18 (18.6)	23 (46.7)	98 (16.4)	

Bold *p*-value means significant difference between zones

^a^carter water seller and at the neighbour

^b^UCA = Urban central area

^c^PCA = Peri-central area

^d^NPA = North peripheral area

^e^SPA = South peripheral area

### Access to water and sanitation

As shown in Table 1, tap water at home was the most commonly used drinking water source (61.7%; *n* = 368), followed by water from public taps (25.5%; *n* = 152), well water (8.6%; *n* = 51) and other sources (4.2%; *n* = 25). In the UCA, 83.4% (*n* = 146) had their own tap water at home, followed by the NPA (64.9%, *n* = 63), PCA (55.8%, *n* = 153) and SPA (12.0%, *n* = 6).

Two thirds of the households (69.6%; *n* = 415) stored drinking water and 16.4% of them (*n* = 98) treated the stored water prior to consumption. Among the households that treated their water prior to storing, 69 (70.4%) used chlorination, 22 (22.4%) used filtration, and 7 (7.1%) employed other methods. Most households (97.3%; *n* = 580) had a toilet facility at home, while the remaining 16 households had no access to sanitation facilities (2.7%). Among households with a toilet, 522 (90.0%) had a septic tank, 222 (38.3%) shared toilet facilities and only 7 (1.2%) households were connected to a sewer. Two hundred and one households (33.7%) reported no kitchen at home. Most of them (63.7%; *n* = 128) prepared their food in the yard of the house, while 18.9% (*n* = 38) prepared food in a covered space provided for cooking in the yard of the house and 17.4% (*n* = 35) in a corridor in the residence.

### Reported prevalence of diarrhoea among children under the age of five

**Fig. 3:**
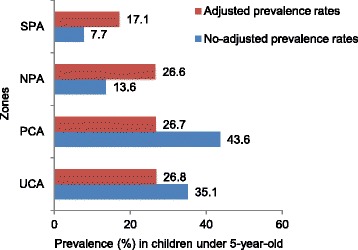
Diarrhoeal prevalence rates by zone before and after adjustment for individual factors in Mbour, Senegal, 2014

**Fig. 4:**
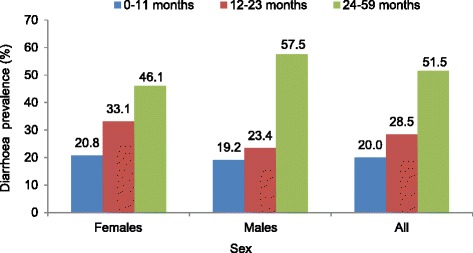
Diarrhoea prevalence among children under 5-years-old by age and gender in Mbour, Senegal, 2014

**Fig. 5:**
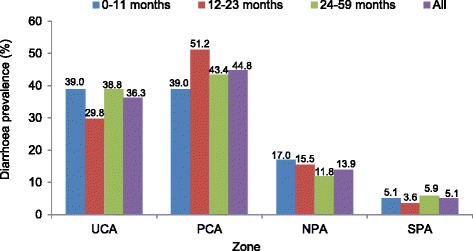
Diarrhoea prevalence among children under 5-year-old by age and zone in Mbour, Senegal, 2014

Diarrhoea prevalence among children under the age of five was estimated based on the number of children who reportedly had diarrhoea during the 2 weeks preceding the interview as the numerator and the overall number of children in the sample as the denominator. Diarrhoeal cases occurring within the 2 weeks preceding the interview were reported for one in four children, giving an overall prevalence of 26.1% (*n* = 295). Prevalence was slightly higher among girls than boys (27.6% and 24.4%, respectively), but this difference was not statistically significant (*P* = 0.22). Adjusted diarrhoea prevalence among children under the age of five did not show a significant difference between zones, with the highest rate observed in the UCA (26.9%) and the lowest rate in the SPA (17.1%). Without adjusting for other variables, the highest diarrhoea prevalence was observed in the PCA (44.8%) and the second highest in the UCA (36.3%), as shown in Figs. 3 and 4. The analysis stratified by age group showed a higher prevalence of diarrhoea in the oldest age group (24–59 months), while the lowest diarrhoea prevalence was observed among children under 12 months (Fig. 3). Diarrhoea prevalence, stratified by age group and zone, is shown in Fig. 5. In this analysis, the highest prevalence was observed among children 12–23 months in the PCA. The highest prevalence among children <12 months was observed in the UCA and NPA. The proportion of children having more than one diarrhoea event during the 2 weeks immediately preceding the survey was 30%.

### Household risk factors associated with diarrhoea

The results from the univariable and multivariable logistic regression analyses are presented in Tables 2 and 3. After adjustment for potential confounders, diarrhoea among children under the age of five was significantly associated with: (i) mother’s unemployment (adjusted (a*OR*) = 1.62, 95% *CI*: 1.18–2.23); (ii) sharing the toilet with other households (a*OR* = 1.69, 95% *CI*: 1.11–2.56); (iii) use of unconventional bag (open bag) for storing household solid waste (a*OR* = 1.75, 95% *CI*: 1.00–3.02); note that solid waste comprises garbage originating from private homes or apartments (also called domestic waste or residential waste); (iv) households with more than one child under the age of five (a*OR* = 2.86, 95% *CI*: 1.70–4.80 and a*OR* = 1.55, 95% *CI*: 1.00–2.40); (v) evacuation of household domestic wastewater in public street (a*OR* = 2.07, 95% *CI*: 1.20–3.55); and (vi) no treatment of stored drinking water (a*OR* = 1.69, 95% *CI*: 1.11–2.56). Conversely, mother’s above 40 years (a*OR* = 0.38, 95% *CI*: 0.22–0.65) and “middle” (a*OR* = 0.64, 95% *CI*: 0.41–0.98) and “richest” (a*OR* = 0.62, 95% *CI*: 0.42–0.90) socioeconomic status were negatively associated with the occurrence of diarrhoea.

**Table 2:** Results of univariate logistic regression for diarrhoea risk factors in Mbour, Senegal, 2014

Two weeks diarrhoea prevalence *N*(total) = 1136 / *N*(cases) = 295		Diarrhoea *N* (%)	Healthy *N* (%)	Univariate logistic regression*		
				*OR*	95% *CI*	*P*-value
**1.Socio-demographic and socioeconomic determinants of diarrhoea**						
Age (in years)	< 25	62 (22.3)	127 (17.1)	Reference		**<0.001**
	25–29	71 (26.7)	159 (21.4)	0.91	0.61–1.38	0.672
	30–39	106 (39.8)	232 (39.3)	0.74	0.51–1.08	0.123
	≥ 40	27 (10.1)	165 (22.2)	0.33	0.20–0.56	**<0.001**
Household’s socioeconomic status	Richest	148 (50.3)	438 (52.1)	1.20	0.85–1.69	0.301
	Middle	58 (19.7)	206 (24.5)	Reference		**>0.053**
	Poorest	88 (30.0)	197 (23.4)	1.58	1.07–2.33	**0.019**
Occupational status	Employed	128 (43.4)	482 (57.3)	Reference		
	Unemployed	167 (56.6)	359 (42.7)	1.75	1.34–2.29	**<0.001**
Marital status	Married	275 (93.5)	765 (91.2)	Reference		0.194
	Unmarried	19 (6.5)	74 (8.8)	0.71	0.42–1.20	0.207
Number of household members	< 5	45 (15.3)	86 (10.2)	1.78	1.17–2.73	**0.008**
	5 to 7	87 (29.5)	224 (26.6)	1.32	0.95–1.85	0.101
	8 to 10	65 (22.0)	197 (23.4)	1.12	0.78–1.61	0.522
	≥ 11	98 (33.2)	334 (39.7)	Reference		**0.049**
Number of children <5 years in the house	< 2	92 (31.2)	177 (21.0)	2.13	1.44–3.15	**<0.001**
	2 to 3	150 (50.8)	447 (53.2)	1.37	0.97–1.95	0.077
	≥ 4	53 (18.0)	217 (25.8	Reference		**<0.001**
**2.Environmental exposure variables associated with diarrhoea**						
Type of toilet in the house	Toilet with pit	254 (88.2)	744 (90.7)	Reference		**0.035**
	Latrine traditional	25 (8.7)	69 (8.4)	1.06	0.66–1.71	0.808
	Sewer network	9 (3.1)	7 (0.8)	3.76	1.38–10.21	**0.009**
Storage of household solid waste	Others**	24 (8.1)	99 (11.8)	Reference		**0.058**
	Pail/basin	104 (35.3)	326 (38.8)	1.32	0.80–2.16	0.279
	Open bag	167 (56.6)	416 (49.4)	1.66	1.02–2.68	**0.040**
Toilet shared with others households	Yes	119 (41.3)	270 (32.9)	1.43	1.09–1.89	**<0.001**
	No	169 (58.68)	550 (67.1)	Reference		**<0.001**
Duration of storage	Two days	38 (20.0)	157 (28.7)	Reference		**<0.001**
	One day	113 (59.4)	290 (53.1)	1.61	1.06–2.44	**0.025**
	Three days	29 (15.3)	64 (11.7)	1.87	1.07–3.29	**0.029**
	More than three days.	10 (5.3)	35 (6.4)	1.18	0.54–2.59	0.689
**3.Behavioural related risk factor for diarrhoea**						
Handwashing after work	No	243 (82.4)	613 (72.9)	Reference		
	Yes	52 (17.6)	228 (27.1)	0.57	0.41–0.80	**<0.001**

Bold significant *p*-value <0.05 derived from the multivariate regression

*Odds ratio (*OR*), confidence interval (*CI*) and *P*-value derived from univariate logistic regression based on likelihood ratio test, overall significant *P*-value of the models are indicated in bold letter. **Others including half metal drum, plastic vacant lost or illegal dumping etc

**Table 3:** Multivariate analysis of risk factors of diarrhoea among children <5 years old in Mbour, Senegal, 2014

Two weeks diarrhoea prevalence *N*(total) = 1136/*N*(cases) = 295		Multivariate logistic regression*		
		a*OR*	95% *CI*	*P*-value
Age (in years)	<25	1.00		
	25–29	0.95	0.62–1.43	0.802
	30–39	0.73	0.46-1.15	0.178
	≥ 40	0.38	0.22–0.65	<﻿0.001**
Occupational status	Employed	1.00		
	Unemployed	1.62	1.18–2.23	0.003**
Household’s socioeconomic status	Richest	0.62	0.42–0.90	0.013**
	Middle	0.64	0.41–0. 98	0.041**
	Poorest	1.00		
Number of children <5 years in the house	<2	2.86	1.70–4.80	<﻿0.001**
	2 to 3	1.55	1.00–2.40	0.035**
	≥ 4	1.00		
Toilet shared with others households	Yes	1.69	1.11–2.56	0.014**
	No	1.00		
Domestic wastewater disposal	Dustbin	1.00		
	Pit	0.96	0.62–1.49	0.868
	Public street	2.07	1.20–3.55	0.009**
Storage of household solid waste	Others	1.00		
	Pail/basin	1.67	0.94–2.97	0.080
	Open bag	1.75	1.00–3.02	0.046**
Treatment of drinking water stored	Yes	1.00		
	No	1.69	1.11–2.56	0.014**

*Adjusted odds ratio (a*OR*), confidence interval (*CI*) and Wald *P*-value in the multivariable mixed regression model including random household intercepts. **﻿Significant *P*-value <0.05 derived from the multivariable regression.The multivariable model was defined including the variables sex, age, educational level, socioeconomic status, number of people per household and number of children under 5 years per household. In addition, all risk factors that had a *P*-value lower than 0.2 in the univariable analyses were included into the multivariable regression models (as indicated in the Table 3)

## Discussion

In this study, we compared diarrhoea prevalence (recall period: 2 weeks) and risk factors among children under the age of five in four zones of Mbour, a medium-size town in Senegal. We found that the 2-week, caregiver-reported prevalence of diarrhoea among children under 5 years was 26%, which is slightly above the rate reported for the same age group in the 2014 Senegalese DHS (19%) [7]. However, our rate is below that reported in the ENSAN 2013 survey report (32%) for the Thiès region [22]. It is also lower than the 35% prevalence among children under 5 years previously reported in Kaédi, Mauritania by Touray and colleagues (2012) [23]. Other studies in secondary cities of sub-Saharan Africa reported lower rates: 23.6% in a 2008 survey in Nouakchott, Mauritania; 14% in 2006 in Yopougon, Côte d’Ivoire; and 13.5% in 2010 in other districts of Nouakchott, Mauritania [23–27]. The high prevalence of diarrhoea in urban Senegal found in the present study was observed in the cold, dry season between February and March, the period of the harmattan, during which most diarrhoea cases and deaths due to rotavirus infection had previously been reported [10]. A study conducted in Burkina Faso during the cold, dry season (December 2009–February 2010) found a rotavirus prevalence of 63.8% among children under the age of five. The same study showed that up to 90% of all diarrhoea cases in this population group were related to rotavirus [28]. In view of these findings, more attention should be given to exploring diarrhoea seasonality and the influence of climatic parameters, in order to more effectively prevent and manage diarrhoea in urban settings in Senegal and elsewhere in sub-Saharan Africa.

According to our study, diarrhoeal prevalence was highest in the PCA (44.8%), which was almost nine times higher than that in the SPA (5.1%). Hence, there is considerable spatial heterogeneity of diarrhoea prevalence, which might partially be explained by differences in the distribution of risk factors across zones, such as living conditions, population density, socioeconomic status and WASH conditions.

We also found that diarrhoea was reported slightly more often among girls compared to boys. In contrast, diarrhoea was more frequent among boys in a study from Sudan [29]. Our finding might be explained by the cultural practices in Senegal, where there is an overt preference for boys over girls that might also affect how mothers or caregivers take care of children. For example, the 2014 Senegalese DHS indicates that care for diarrhoea concerns was sought more frequently for boys (36%) than for girls (29%) [8]. This suggests that boys suffer more frequently from diarrhoea compared to girls, unless there is a tendency to take girls to the doctor less often.

In our study, the prevalence of diarrhoea was highest in the age group 24–59 months (51.5%). This finding is in contrast to results from a study in Burkina Faso, where children under the age of 12 months had the highest rate of diarrhoea (44%) [28, 30]. However, in the urban slums of Senegal, children aged 2–5 years are likely to have a high risk of exposure to diarrhoeal pathogens because they have considerable independence. They are often highly mobile and play unsupervised within the community environment, where there is a high level of contamination.

Our study showed that the risk of diarrhoea was significantly associated with the mother’s occupation (i.e. housewife was associated with higher diarrhoea risk compared to those working in the private or public sector). This finding is consistent with other reports, which found that parental occupation was associated with diarrhoeal occurrence [31, 32]. Although socioeconomic status (middle and poorest) showed a significant association with the occurrence of diarrhoea in the bivariate analysis, it was not significant when other variables were included. In the multivariate analyses, we found that children living in better off households were less likely to have diarrhoea compared to their lower wealth counterparts. A likely explanation of this observation is that wealth is associated with better access to household amenities and facilities, including those related to better hygiene and environmental health, which might reduce the risk of diarrhoea. In addition, wealth allows parents to use health services more frequently [33, 34]. However, Root suggested that wealthy parents may be unable to reduce the risk of diarrhoea due to factors beyond their control, such as contaminated community environment or lack of water [35]. However, many other studies indicate that socioeconomic factors are strongly associated with the occurrence of diarrhoea; this appears to confirm the social determinants of health [36, 37].

The presence of two or more children under the age of five living in the same household was significantly associated with the occurrence of diarrhoea. This observation is consistent with a number of cross-sectional studies conducted in Nigeria and Cameroon [38–40]. These findings indicate that a large number of children residing in the same household is a predictor of diarrhoea among children under the age of five. More than two children in this age range in a single household probably means that contact with potential pathogens is more frequent than in households with only one or two young children [38]. This difference could be due to the challenges of taking care of multiple young children. It follows, then, that longer spacing between births and exclusive breastfeeding in the first 2 years of life might have a positive impact on diarrhoea prevention.

We did not find a significant association between drinking water sources and the occurrence of diarrhoea. This might be explained by the very small differences across the sampled households in terms of drinking water sources. In urban Africa, there are multiple sources of drinking water (e.g. well, tap water at home and public taps). Even if a household has a water connection at home, an inhabitant might need to go to the public tap or use well water due to recurrent cuts in the network. A study in southwest Ethiopia did not find a significant association between drinking water sources and the risk of diarrhoea either [41]. In contrast, two different studies from Ethiopia found that water sources are an important environmental predictor of diarrhoea morbidity [42, 43]. We found that the lack of treatment of stored drinking water was positively associated with the prevalence of diarrhoea.

We found that diarrhoea occurrence was not significantly associated with the presence of a toilet. This finding is in line with a recent study from Ethiopia, where no association was found between sanitary facilities and the occurrence of diarrhoea [41]. Conversely, another study from Ethiopia found that the availability of a latrine was negatively associated with diarrhoea after controlling for potential confounding factors [44]. The type of toilet showed a significant association with the occurrence of diarrhoea in our bivariate analyses. Rather unexpectedly, the association became insignificant when other variables were included in the multivariate analyses. We found that sharing a toilet with other households was associated with a high risk of diarrhoea.

Our study showed that the use of open bags for storing household solid waste was significantly associated with the prevalence of diarrhoea. In a study carried out in Ibadan, Nigeria, indiscriminate disposal of solid waste was significantly associated with a high rate of diarrhoea [45]. Studies in Ethiopia also revealed that open disposal of waste around the house was a risk factor for diarrhoea [26, 46]. We also found that evacuation of domestic wastewater from households into public streets was significantly associated with the risk of diarrhoea. The likely explanation for these results is that inappropriate disposal of solid waste and evacuation of wastewater in public streets create breeding sites for insects, which may spread diarrhoeal pathogens from the open waste to water or food.

Evacuation of human excreta into septic tanks conferred an increased risk of diarrhoea but these associations were not statistically significant, which may be due to its rare occurrence. We also found that mother’s age (i.e. 40 years and above) significantly reduced the risk of children’s diarrhoea. This finding might be explained by greater experience in childcare, hygiene and feeding practices with age.

Our findings support the ‘urban health penalty’ hypothesis, which posits that the poor in urban areas are pushed to marginal areas, where environmental health conditions are not suitable for health. This issue is particularly pronounced in secondary cities, where access to clean water and improved sanitation, and socioeconomic conditions more broadly, have been compromised by populations moving into urban areas that are unregulated and poorly managed. Such conditions might result in urban slums that are characterised by inadequate safe water supply, lack of drainage and sewage networks and the absence of sanitation and solid waste removal.

### Limitations

Our study has several limitations that are offered for consideration. First, we pursued a cross-sectional survey; hence, caution is required regarding causality, as the results presented here are related to the cold, dry season (February and March). To better understand seasonal variability, we are in the process of conducting a survey in the same area in the hot, wet season (July and August). Second, the assessment of diarrhoea prevalence was based on caregivers’ reports, which may have introduced some recall bias, despite the relatively short recall period of 2 weeks. Third, we did not undertake microbiological analysis of stool samples and drinking water samples, mainly due to budget limitations. Microbiological analysis of drinking water at source and household levels will be conducted in subsequent follow-up studies in the study area.

Despite these limitations, our study provides new insight into the extent of diarrhoea among children under five in Mbour. Our results might be helpful for designing appropriate interventions for preventing childhood diarrhoea in the study area.

## Conclusions

Our study showed that the reported prevalence of diarrhoea among children under the age of five is high in Mbour (26%), which is similar to previous estimates obtained from DHS. The disaggregated findings provide a useful baseline for more targeted interventions and future studies in more vulnerable urban settings in the area. The study indicates that there is a need for effective preventive measures to reduce the high prevalence of diarrhoea in secondary cities in Senegal. Health intervention programmes, including increasing priority of solid waste and wastewater management, should be introduced and tested, particularly in the PCA and UCA, where diarrhoeal diseases are most common, in order to reduce the prevalence and burden of diarrhoea. The findings also provide useful information to the existing national programme for the fight against diarrhoea and to all other actors developing targeted interventions for preventing childhood diarrhoea.

## Additional file

Additional file 1 Multilingual abstracts in the five official working languages of the United Nations. (PDF 749 kb)

## Abbreviations

ANSD: National Agency of Statistics and Demography
a*OR*: Adjusted odds ratio
*CI*: Confidence interval
DALY: Disability-adjusted life year
DHS: Demographic and Health Survey
ENSAN: National Survey on Food Security and Nutrition
NPA: North peripheral area
*OR*: Odds ratio
PCA: Pericentral area
SPA: South peripheral area
UCA: Urban central area
WASH: water, sanitation and hygiene
WHO: World Health Organization
