# Supplementary material for: Prevalence of diarrhoea and risk factors among children under five years old in Mbour, Senegal: a cross-sectional study
Source: Infect Dis Poverty. 2017 Jul 6;6:109. doi: 10.1186/s40249-017-0323-1 (PMC5499039; doi:10.1186/s40249-017-0323-1)
Supplement: Additional file 1 — Multilingual abstracts in the five official working languages of the United Nations. (PDF 749 kb) [file 40249_2017_323_MOESM1_ESM.pdf]

Translation of the abstract into the five official working languages of the United Nations

انتشار الإسهال وعوامل الخطر بين الأطفال دون سن الخامسة في مبور، السنغال:دراسة مقطعية

سوكنا تيام، أميناتا ن ديين، صموئيل فوريمان، ميركو س. وينكلر، إبراهيما سي، جاك أ. نديوني، كريستيان شندلر، بينيلوب فوناتسو، يورج أوتزنجر، عثمان فاي وجويلاديو سيسي

ملخص

خلفية: لا تزال أمراض الإسهال أحد أهم أسباب الوفيات والمراضة بين الأطفال، ولا سيما في البلدان المنخفضة والمتوسطة الدخل. في السنغال، الإسهال هو المسؤول عن 15٪ من مجموع وفيات الأطفال تحت سن الخامسة وهو ثالث سبب رئيسي لوفيات الطفولة. لتخطيط وتنفيذ استراتيجيات وقائية محددة الأهداف، هناك حاجة لفهم سياق محدد من محددات أمراض الإسهال. وكان الهدف من هذه الدراسة تحديد عوامل الخطر لأمراض الإسهال في الأطفال دون سن الخامسة في مبور، السنغال.

أساليب: بين شهري فبراير ومارس عام 2014، أجري مسح مقطعي في أربع مناطق من مبور لتقدير عبء أمراض الإسهال (أي نوبات الإسهال في الأسبوعين السابقين للمسح) وعوامل الخطر المرتبطة بها. كانت المناطق المغطاة هي المناطق الوسطى وشبه الوسطى وشمال الطرفية وجنوب الطرفية الحضرية. وعموما، تم مسح 596 أسرة من خلال استبيان، حيث جُمعت معلومات عن العوامل السلوكية الاجتماعية والديموجرافية والبيئية والصحية. استخدمت تحليلات الانحدار اللوجستي أحادية المتغيرومتعددة المتغيرات لتحديد عوامل الخطر المرتبطة بحدوث الإسهال.

النتائج: كانت نسبة الإصابات المبلغ عنها من الإسهال بين الأطفال دون سن الخامسة خلال الأسبوعين السابقين للمسح 26٪. دون تعديل، لوحظ أن أعلى معدلات انتشار الإسهال كانت في المناطق القريبة من وسط (44.8٪) والمناطق المركزية الحضرية (36.3٪). كشف الانحدار متعدد المتغيرات وجود ارتباطات ذات دلالة إحصائية بين أمراض الإسهال وبطالة الأمهات (نسبة الأرجحية المعدلة [aOR] = 1.62، فاصل الثقة 95٪ [CI] 1،18-2،23)، واستخدام أكياس مفتوحة لتخزين النفايات المنزلية (أو = 1.75، 95٪ CI 1،00-3،02)، إلقاء النفايات المنزلية في الشوارع العامة (أو = 2.07، 95٪ CI 1،20-3،55)، عدم معالجة مياه الشرب المخزنة (aOR = 1.69، 95٪ CI 1،11-2،56) واستخدام المراحيض المشتركة (أو = 1.69، 95٪ CI 1،11-2،56).

استنتاج: لقد وجدنا ارتفاع في معدل انتشار الإسهال بين الأطفال دون سن الخامسة في مبور، وكانت أعلى نسبة انتشار في المناطق الوسطى وشبه الوسطى. هذه النتائج تؤكد على الحاجة إلى تدخلات الصحة العامة لتخفيف عبء الإسهال بين الفئات الضعيفة. ينبغي تشجيع التخلص من النفايات الصلبة والحد من التعرض لمياه الصرف الصحي دون تأخير.

Translated from English version into Arabic by Mahmoud Sami, through

TRANSLATORS
WITHOUT BORDERS

塞内加尔 Mbour 地区 5 岁以下儿童腹泻情况及危险因素：一项横断面研究

Sokhna Thiam, Aminata N. Diène, Samuel Fuhrimann, Mirko S. Winkler, Ibrahima Sy, Jacques A. Ndione, Christian Schindler, Penelope Vounatsou, Jürg Utzinger, Ousmane Faye and Guéladio Cissé

摘要

引言：腹泻仍然是中、低收入国家儿童致病和致死的重要原因。在塞内加尔 5 岁以下儿童死亡中 15%由腹泻所致，也因此成为儿童第 3 大死因。为针对性地制定规划以及实施防治策

略，需要掌握特定背景下导致腹泻的因素。本研究的目的就是寻找塞内加尔 Mbour 地区 5 岁以下儿童的腹泻危险因素。

方法：2014 年 2-3 月，在 Mbour 的四个区开展了一项横断面调查以评估腹泻（腹泻定义为调查前 2 周内发生过腹泻）的疾病负担和相关危险因素。调查地区涉及城中区、城中周边郊区、北部郊区和南部郊区。共对 596 户进行了问卷调查，收集的信息包括社会人口学、环境和卫生及行为因素。采用单因素和多因素 logistic 回归分析鉴别腹泻的相关危险因素。

结果：调查前 2 周内 5 岁以下儿童的腹泻发生率为 26%。未校正前，腹泻发生率率最高的地区是城中周边郊区(44.8%)和城中区(36.3%)。多因素回归分析表明腹泻与下述因素显著相关，即母亲失业（校正的 *OR*（a*OR*）：1.62；95% 置信区间[*CI*]：1.18 – 2.23）、使用无盖桶储存家庭垃圾 （a*OR*：1.75；95% *CI* ：1.00 – 3.02)、在公共街道上堆放家庭垃圾放 （a*OR*：2.07；95% *CI*：1.20 – 3.55)、储存饮用水不处理 （a*OR*：1.69；95% *CI*：1.11–2.56) 以及共用厕所 (a*OR* = 1.69；95% *CI*：1.11–2.56)。

结论：我们发现在 Mbour 地区 5 岁以下儿童腹泻发生率很高，其中以城中区和城中周边郊区为最。这些发现强调需要开展公共卫生干预以缓解脆弱人群的腹泻疾病负担。应该立即开展措施以促进固体垃圾处理和减少废水暴露。

Translated from English version into Chinese by Men-Bao Qian, through
CHINESE CENTER FOR DISEASE CONTROL AND PREVENTION
NATIONAL INSTITUTE OF PARASITIC DISEASES

La prévalence de la diarrhée et les facteurs de risque chez les enfants de moins de cinq ans à Mbour, au Sénégal: Une étude transversale

Sokhna Thiam, Aminata N. Diène, Samuel Fuhrimann, Mirko S. Winkler, Ibrahima Sy, Jacques A. Ndione, Christian Schindler, Penelope Vounatsou, Jürg Utzinger, Ousmane Faye et Guéladio Cissé

Résumé

Contexte: Les maladies diarrhéiques restent une cause importante de mortalité et de morbidité chez les enfants, en particulier dans les pays à faible revenu et à revenu intermédiaire. Au Sénégal, la diarrhée est à l'origine de 15% de tous les décès chez les enfants de moins de cinq ans et est la troisième principale cause de mortalité infantile. Pour une planification et une mise en œuvre de stratégies de prévention adaptées au contexte, il est nécessaire de comprendre les causes des maladies diarrhéiques. Le but de cette étude est d'identifier les facteurs de risque des maladies diarrhéiques chez les enfants de moins de cinq ans à Mbour, Sénégal.

Méthodes: entre février et mars 2014, une étude transversale a été menée dans quatre zones de Mbour pour estimer le fardeau des maladies diarrhéiques (c'est-à-dire les épisodes de diarrhée dans les deux semaines précédant l'enquête) et les facteurs de risque associés. Les zones couvertes sont les zones urbaines centrale, du péri-centre, de la périphérie du nord et du sud. Dans l'ensemble, 596 ménages ont répondu au questionnaire qui nous a donné des informations sur les facteurs comportementaux sociodémographiques, environnementaux et l'hygiène. Les analyses de régression logistique univariées et multivariées ont été utilisés pour identifier les facteurs de risque associés à l'apparition de la diarrhée.

Résultats: Le taux de prévalence de la diarrhée chez les enfants de moins de cinq ans au cours des deux semaines précédant l'enquête était de 26%. Sans ajustement, les taux de prévalence de la diarrhée les plus élevés ont été observés dans la péri-centrale avec 44,8% et les zones urbaines centrales avec 36,3%. En ce qui concerne la régression multivariée, elle a révélé des corrélations significatives entre les maladies diarrhéiques et le chômage des mères (odds ratio ajusté [a*OR*] = 1,62, soit 95% d'intervalle de confiance [*IC*] 1,18 à 2,23), l'utilisation de sacs ouverts pour le stockage des déchets ménagers (un*OR* = 1,75 soit 95% *CI* 1,00 à 3,02), l'évacuation des déchets ménagers sur la voie publique (un*OR* = 2,07, IC à 95% 1,20 à 3,55), l'absence de moyens de traitement de l'eau potable stockée (a*OU* = 1.69, 95% *IC* 1,11 à 2,56) et l'utilisation de toilettes communes (un*OR* = 1,69 soit 95% *IC* 1,11-2,56).
Conclusion: Nous avons constaté une forte prévalence de la diarrhée chez les enfants de moins de cinq ans à Mbour, avec la plus forte prévalence se produisant dans les zones centrales et péri-centrales. Ces résultats soulignent la nécessité de mener des interventions de santé publique pour alléger le fardeau de la diarrhée chez les groupes vulnérables. La promotion de l'élimination des déchets solides et la réduction de l'exposition aux eaux usées devrait être mis en œuvre sans délai.

Translated from English version into French by Simonyetna, through

TRANSLATORS
WITHOUT BORDERS

Кросс-секционное исследование распространенности диареи и факторов риска среди детей в возрасте до пяти лет в Мбуре (Сенегал)

Sokhna Thiam, Aminata N. Diène, Samuel Fuhrimann, Mirko S. Winkler, Ibrahima Sy, Jacques A. Ndione, Christian Schindler, Penelope Vounatsou, Jürg Utzinger, Ousmane Faye and Guéladio Cissé

Аннотация

Базовая проблематика. Диарейные заболевания остаются важной причиной заболеваемости и смертности среди детей особенно в странах с низким и средним уровнями дохода. В Сенегале диарея приводит к 15% всех случаев смерти среди детей в возрасте до пяти лет и является третьей ведущей причиной детской смертности. Для целевого планирования и реализации стратегий по предупреждению необходимо контекстно-специфическое понимание детерминант диарейных заболеваний. Цель данного исследования состояла в том, чтобы определить факторы риска развития диарейных заболеваний у детей в возрасте до пяти лет в Мбуре (Сенегал).
Методы.В четырех зонах города Мбура в период с февраля по март 2014 года было проведено кросс-секционное исследование для оценки бремени диарейных заболеваний (т. е. эпизодов диареи в течение двух недель, предшествовавших исследованию) и связанных с ними факторов риска. Зоны включали в себя городской центральный район, районы, располагающиеся вокруг центрального, а также северный и южный периферийные районы.

В целом было опрошено 596 домохозяйств с использованием вопросника, что дало информацию о социально-демографических, экологических и гигиенических поведенческих факторах. Для выявления факторов риска, связанных с возникновением диареи, использовались однофакторный анализ и многопараметрическая логистическая регрессия.
Результаты. Распространенность диареи среди детей в возрасте до пяти лет в течение двух недель до проведения исследования составляла 26%. Самые высокие показатели распространенности диареи наблюдались в зонах вокруг центра (44,8%) и городских центральных зонах (36,3%) без применения каких-либо корректировок. С помощью многопараметрической регрессии выявлены существенные связи между диарейными заболеваниями и безработицей матерей (скорректированное отношение шансов [*сОШ*] = 1,62, 95% доверительного интервала [*ДИ*] 1,18–2,23), использованием открытых мешков для хранения бытовых отходов (*сОШ* = 1,75, 95% *ДИ* 1,00–3,02), эвакуацией бытовых отходов на улицы (*сОШ* = 2,07, 95% ДИ 1,20–3,55), отсутствием обработки хранимой питьевой воды (*сОШ* = 1,69, 95% *ДИ* 1,11–2,56) и использованием общих туалетов (*сОШ* = 1,69, 95% *ДИ* 1,11–2,56).
Вывод. Мы обнаружили высокий уровень распространенности диареи у детей в возрасте до пяти лет в Мбуре при самом высоком показателе распространенности в центральном и расположенных вокруг центрального районах. Эти результаты подчеркивают необходимость принятия мер в области здравоохранения, чтобы облегчить бремя диареи среди уязвимых групп населения. Необходимо безотлагательно принимать меры по содействию утилизации твердых отходов и сокращению воздействия сточных вод.

Translated from English version into Russian by Anna Romanenko, through

TRANSLATORS
WITHOUT BORDERS

Prevalencia de la diarrea y factores de riesgo en los niños menores de cinco años en Mbour, Senegal: un estudio transversal

Sokhna Thiam, Aminata N. Diène, Samuel Fuhrimann, Mirko S. Winkler, Ibrahima Sy, Jacques A. Ndione, Christian Schindler, Penélope Vounatsou, Jürg Utzinger, Ousmane Faye y Guéladio Cissé

Resumen

Antecedentes: las enfermedades diarreicas siguen siendo una causa importante de mortalidad y morbilidad en niños, sobre todo en países de ingresos bajos y medios. En Senegal, la diarrea es la causante del 15% de las muertes en niños menores de cinco años y es la tercera causa principal de muerte infantil. Para lograr una planificación focalizada y la implementación de estrategias de prevención, es necesario comprender los determinantes de las enfermedades diarreicas dentro de cada contexto específico. El objetivo de este estudio fue identificar los factores de riesgo de las enfermedades diarreicas en niños menores de cinco años en Mbour, Senegal.

Métodos: entre febrero y marzo de 2014, se llevó a cabo una encuesta transversal en cuatro zonas de Mbour, a fin calcular la carga de las enfermedades diarreicas (es decir, los episodios de diarrea que tuvieron lugar en las dos semanas anteriores a la encuesta) y los factores de riesgo asociados. Las zonas cubiertas fueron las áreas urbanas centrales, pericentrales y periféricas del norte y sur. En líneas generales, se encuestaron 596 hogares que proporcionaron información sobre factores sociodemográficos, ambientales y de comportamientos de higiene. Se utilizaron análisis de regresión logística univariados y multivariados para identificar los factores de riesgo asociados con la incidencia de diarrea.

Resultados: se registró una prevalencia de diarrea del 26% en niños menores de cinco años en las dos semanas anteriores a la encuesta. Sin efectuar ajustes, se observaron mayores tasas de prevalencia de diarrea en las zonas pericentrales (44,8%) y en las zonas urbanas centrales (36,3%). La regresión multivariada reveló asociaciones significativas entre las enfermedades diarreicas y la falta de empleo de las madres (razón de probabilidades ajustadas [a*OR*] = 1,62; intervalo de confianza [*CI*] del 95%: 1,18 - 2,23); el uso de bolsas abiertas para el almacenamiento de residuos domésticos (a*OR* = 1,75;*CI* del 95%: 1,00 - 3,02), la eliminación de residuos domésticos en la vía pública (a*OR* = 2,07; CI del 95%: 1,20 - 3,55), la falta de tratamiento de agua potable almacenada (a*OR* = 1,69; *CI* del 95%: 1,11 - 2,56) y el uso de baños compartidos (a*OR* = 1,69; *CI* del 95%: 1,11 a 2,56).

Conclusión: encontramos que la diarrea se presenta de forma predominante en niños menores de cinco años en Mbour, y que la mayor prevalencia se produce en las áreas centrales y pericentrales. Estos resultados destacan la necesidad de llevar a cabo intervenciones de salud pública para aliviar la carga de la diarrea en grupos vulnerables. Es necesario implementar de forma inmediata la promoción de la eliminación de residuos sólidos y la reducción de la exposición a las aguas residuales.

Translated from English version into Spanish by María Paula Gorgone, through

TRANSLATORS
WITHOUT BORDERS
